# Canine Rise Method: A Conservative Approach for Worn Teeth Rehabilitation with Different Adhesive Restorative Materials

**DOI:** 10.1155/2022/9949879

**Published:** 2022-02-27

**Authors:** Hamid Kermanshah, Aws Alzwghaibi, Maitham Al-Tufaili, Sholeh Ghabraei

**Affiliations:** ^1^Dental Research Center, Dentistry Research Institute, Department of Restorative Dentistry, School of Dentistry, Tehran University of Medical Sciences, Tehran, Iran; ^2^Department of Orthodontics, School of Dentistry, Shahid Beheshti University of Medical Sciences, Tehran, Iran; ^3^Department of Restorative Dentistry, School of Dentistry, Tehran University of Medical Sciences, Tehran, Iran; ^4^Department of Endodontics, School of Dentistry, Tehran University of Medical Sciences, Tehran, Iran

## Abstract

**Background:**

In this article, two cases with generalized dental wear and four cases with localized dental wear are reported. In some of the cases, the worn teeth were restored with direct composite techniques with no mock-up and articulator mounting according to the canine rise method. This method is used without any change in the intercuspation of posterior teeth without a muscle relaxation appliance. It introduces a stable occlusal condition that can alter neuromuscular reflex activity, leading to improvements in certain muscle pain disorders. It is also used to protect the teeth and supporting structures from abnormal forces that might create a further breakdown and/or tooth wear.

**Methods:**

The first step of treatment in all the cases was a composite build-up for maxillary and mandibular canines to restrict and guide the horizontal and vertical jaw movements and create adequate space for restorations. The second step was the placement of direct or indirect restorations on upper and lower anterior teeth efficiently and accurately.

**Results:**

This method enhanced the esthetic outcomes in a conservative approach with no reduction in tooth material or time-consuming treatments.

**Conclusions:**

This technique can be suggested for the treatment of worn teeth in patients with Angle's Cl I and Cl II classifications of malocclusion based on the follow-up results. It is not applicable for the treatment of worn teeth in patients with Angle's class III because occlusion is reversed, and canine teeth do not have guidance role. *Practical Implications*. This method significantly reduces the overall treatment time, and additional steps are required to restore the worn-out teeth and/or occlusion using the canine rise method.

## 1. Introduction

One of the well-established occlusal diseases (masticatory dysfunction) is dental wear. It is one of the common destructive dental disorders due to several etiological factors. Usually, this disease is progressive without intervention, and the eventual outcome is tooth loss [1]. Dawson, a pioneer in occlusal disease, has described the condition as follows: the process resulting in the noticeable loss of the occluding surfaces of the teeth [2]. However, Dowson has defined it as “deformation or disturbance of function of any structures within the masticatory system that are in disequilibrium with a harmonious interrelationship between the TMJs, the masticatory musculature, and the occluding surfaces of the teeth [2]. Therefore, we can draw a simple conclusion that dental wear is associated with neuromuscular and/or TMJ disorders.

Crown length and tooth general appearance changes are caused by a multifactorial disease; dental wear, attrition, erosion, and abrasion are the responsible factors [3, 4]. Generally, dental wear is associated with several biological, functional, and esthetic problems [5].

Tooth wear is a significant dental problem caused by various factors, including mechanical, physical, chemical, or trauma. Based on the Adult Dental Health Survey (ADHS), tooth wear is found in approximately 77% of adults, with 15% exhibiting moderate wear and 2% having severe tooth wear [6]. Loomans et al. defined severe tooth wear as “tooth wear with substantial loss of tooth structure, with dentin exposure and significant loss (≥1/3) of the clinical crown” [1], while pathological tooth wear was defined as “tooth wear which is atypical for the age of the patient, causing pain or discomfort, functional problems, or deterioration of esthetic appearance,” which if it progresses, might give rise to undesirable complications of increasing complexity [1].

Thornton defined canine guidance as disocclusion of all the teeth by the contact of unilateral maxillary and mandibular canines only in lateral excursion movement; it is also known as “canine-protected occlusion, mutually protected occlusion, canine disocclusion, canine-lift, and canine rise” [7]. Canine rise occlusion, compared to the group function occlusion, is more efficient in reducing tooth surface friction. Another advantage of canine rise occlusion is the reduction of masticatory muscle activities. Many electromyography (EMG) studies [8–11] and intraoral bite splint studies [12–15] have shown that canine rise occlusion leads to more reductions in masticatory muscle activity compared to the group function occlusion. Numerous clinical studies have indicated that the lack of canine guidance can damage the dentition [16]. The strategic location of the canines away from the fulcrum was effective in reducing compression on the TMJ [10].

Since one of the mechanisms for the occlusal disease is friction, [2] reducing the friction on the tooth surface can be a strategy to control it. It seems that the involvement of the incisal edge of canine teeth in lateral excursion movement reduces friction; therefore, a reduction in tooth wear is expected. Canine teeth are an excellent choice for decreasing tooth occlusal friction because of their dome-shaped morphology. New technologies and advances nowadays allow us to use composites to treat dental wear since the technology of dental adhesives has had an accountable advancement in recent years. Several studies have shown the accountability of composite restorations as a treatment choice for worn teeth with acceptable results [17, 18]. A systematic review of treatment options by Muts et al. recommended conventional ceramic restorations and composite resins as a choice for generalized dental wear treatment [5]. The use of direct composite restorations is conservative and relatively easy to maintain compared to other applicable restorative techniques for dental wear [19].

Minimally invasive treatment strategies are recommended according to the dynamic restorative treatment concept, which is based on new guidelines regarding the treatment of patients with severe dental wear [1]. The present report presents six cases of dental wear treated by the canine rise method. The treatment plan for all the cases was based on adhesive, conservative, and esthetic principles. The first stage of treatment was building up the maxillary and mandibular canines by composite restorations without the mock-up and articulator stages, as mentioned before. It was carried out to restrict and guide the horizontal and vertical jaw movements and create adequate space between the upper and lower incisors, while the posterior teeth are still in contact. Therefore, the mandible can move backward to the centric relation position without increasing the vertical dimension.

## 2. Cases Presentation

### 2.1. Case 1

**Figure 1:**
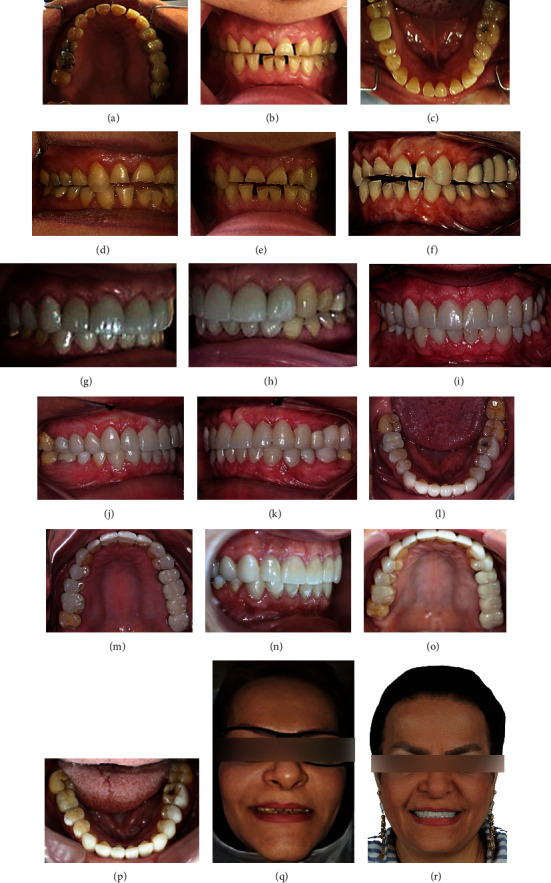
The first case of generalized dental wear. (a)–(c), intraoral view before treatment; (b), frontal view; (d), (e), and (f) show canine rise approach to treat this case using composite resin on upper and lower canines. Definitive restorations (first step) for upper and lower teeth after canine rise approach shown in (g) and (h). Definitive restorations (second step) for veneering upper and lower teeth except some posterior teeth shown in (i), (j), and (k) frontal and lateral views; (l) and (m), occlusal views. Follow-up after three years of initial treatment shown in (n), (o), and (p) occlusal and frontal views. (q) and (r) show facial views before and after treatment.

The patient suffered from generalized severe dental wear involving both the anterior and posterior teeth, as seen in Figures 1(a)–1(c). Her chief complaint involved esthetic problems. The teeth were too short and discolored with a diastema between them. The first stage of treatment was started by composite restoration for maxillary and after mandibular canines with direct restorative technique without any need for mock-up, wax-up, and an articulator to restrict and guide the horizontal jaw movements. The canine teeth were built up to the original shape after two weeks' recall, and the restriction of jaw movements, relaxation of the masticatory muscles, and rotation and backward movement of the mandible to centric relation were achieved. Therefore, adequate occlusal space between maxillary and mandibular anterior teeth was created with the same intercuspation of posterior teeth, as seen in Figures 1(d)–1(f). As shown in Figures 1(d)–1(f), the contact between 17, 47, 27, and 37 (FDI tooth numbering system), confirms that space has been created with the same intercuspation of posterior teeth. At the two weeks' recall appointment, the impression of maxillary and mandibular teeth was taken to mock up and construct provisional composite restorations. After a few weeks, the patient was evaluated for esthetic, phonetic, and function before definitive treatment. The patient was not satisfied with the color of the teeth; therefore, the treatment plan was shifted to laminate veneers. The first step was to place indirect ceramic veneers (IPS e.max Press, Ivoclar Vivadent) on 11, 12, 13, 21, and 22, as seen in Figures 1(g) and 1(h). Then, indirect ceramic veneers were placed on 23. After restoring the anterior guidance of the patient, restorations of posterior teeth (except the second molars) were constructed by conservative indirect ceramic veneers or a porcelain-fused-to-metal bridge on 25, 26, 27, and 28 or crown on 46, as seen in Figures 1(i)–1(m). In this case, the canine rise method was used by indirect restorations. The follow-up photography, after three years, is presented in Figures 1(n)–1(p); while, Figures 1(q) and 1(r) showed the case before and after treatment, respectively.

### 2.2. Case 2

**Figure 2:**
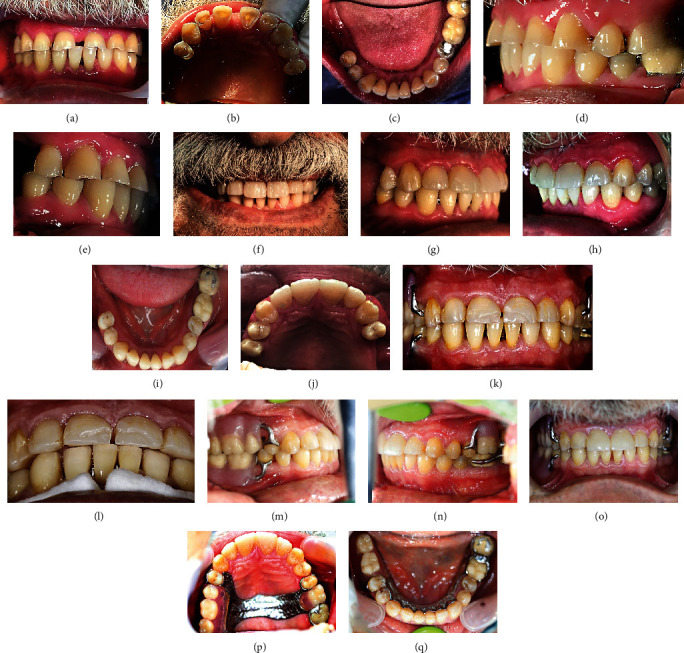
The second case of generalized dental wear; before treatment: (a), Frontal view; (b) and (c), occlusal views; (d) and (e), lateral views. After treatment: (f), facial view; (g) and (h), lateral views; (i) and (j), occlusal views of upper and lower dental arches. (k) and (l) show this case six months after treatment; there was a fracture of restorations on maxillary central incisors before constructing upper and lower removable partial dentures. Follow-up one year after treatment; (m) and (n), lateral views; (o), frontal view; (p) and (q), occlusal view of upper and lower jaws. Note: there was a class II disto-occlusal caries in mandibular left first premolar one year after treatment in the last image (q).

The patient suffered from generalized severe dental wear involving both the anterior and posterior teeth, as seen in Figures 2(a)–2(e). The chief complaint was the poor esthetic appearance of the anterior teeth and tooth sensitivity. Based on clinical and radiographic examinations of worn teeth, there was no evidence of necrosis or irreversible pulpitis. Thus, the conservative approach, “canine rise method,” was adopted through a direct restorative technique without mock-up. First, a build-up by composite restorations was carried out for 13, 23, 33, and 43 to restrict and guide the horizontal and vertical jaw movements and create an adequate space for direct restorations. Isolation was achieved by inserting a rubber dam. Selective etching with 40% phosphoric acid was used for 20 seconds, and an adhesive agent (G-Premio BOND, GC) was placed; then, composite resin (Essentia, GC) build-up was carried out using the incremental technique. During three appointments, the composite was added only to the attrition sites of all the teeth, and the diastema between 11 and 21 was completely closed with micro- or nanohybrid composite resin material (Essentia Medium Dentin (MD) and dark enamel (DE), GC) without any preparation as seen in Figures 2(f)–2(j). The patient was re-evaluated after six months. In clinical examination, chipping in composite restorations of 11 and 21 was observed, as shown in Figures 2(k) and 2(l). Therefore, composite defects were repaired. The next step was to construct upper and lower removable partial dentures, as seen in Figures 2(m)–2(p). In clinical follow-up after one year, caries (class II disto-occlusal) on 34 was treated by a composite restoration, as seen in Figure 2(q). This case is an example of generalized tooth wear with full-mouth rehabilitation by direct composite restorations based on the canine rise method.

### 2.3. Cases 3–6

**Figure 3:**
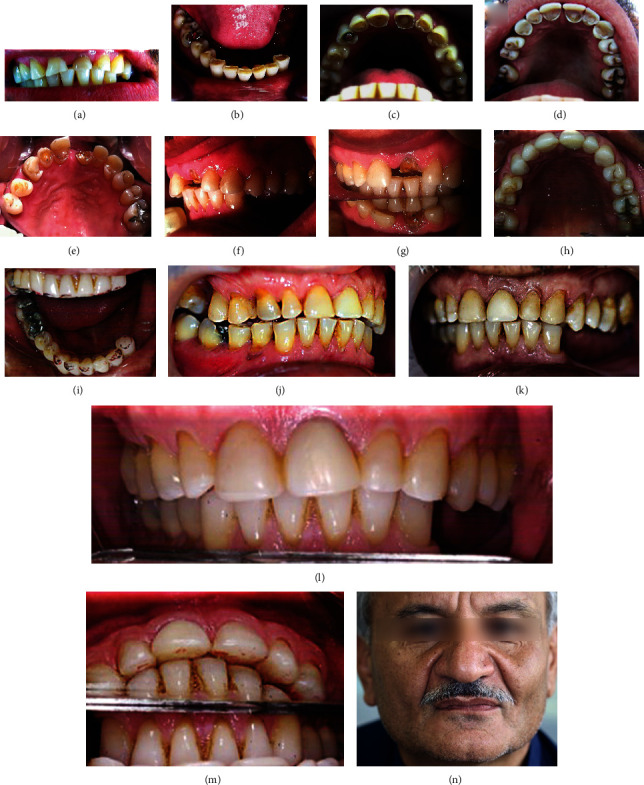
First case of localized dental wear. (a)–(c), intraoral and frontal views before treatment; (d), dental wear treatment without considering canine rise method by direct composite restoration seven years ago; (e)–(g), fracture of maxillary left central incisor two years after initial treatment. Therefore, this case was retreated by composite resin with the canine rise method as shown in (h) and (i). The backward position of the mandible is confirmed by comparing (f) and (g). The follow-up five years after initial dental wear treatment by direct composite resin with canine rise method is shown in (j)–(l), intraoral lateral and frontal views; (m) shows the relationship between upper and lower anterior teeth; (n), extraoral facial view.

**Figure 4:**
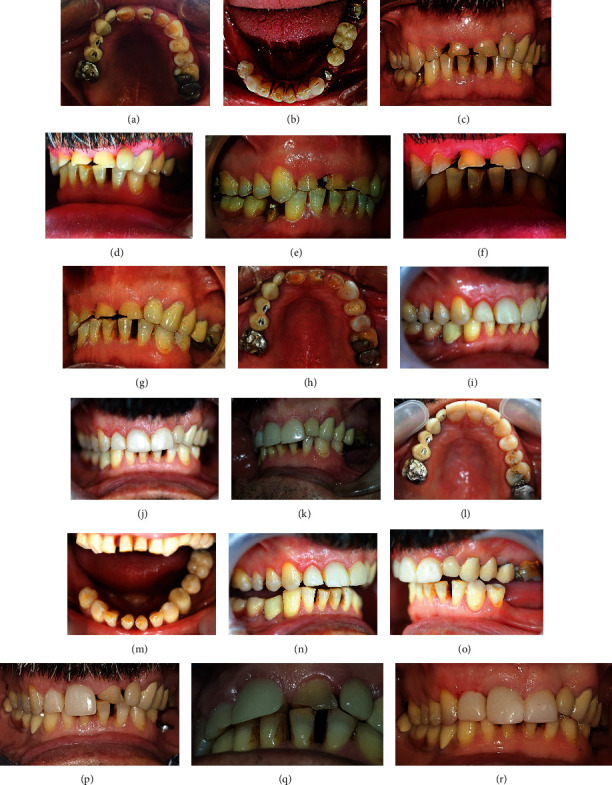
The second case of localized dental wear: (a)–(d), occlusal and frontal views before treatment; (e)–(h), frontal, lateral, and occlusal views after the canine rise method and creation of space for the restorative material. After 1.5 years of definitive treatment, (i)–(k), frontal and lateral views; (l) and (m), occlusal views. The lateral excursion is showed with canine rising (n and o). After 2 years, composite restoration of left central incisor was completely debonded, and the created space by the procedure is obvious in (p) and (q). New composite restoration (r).

**Figure 5:**
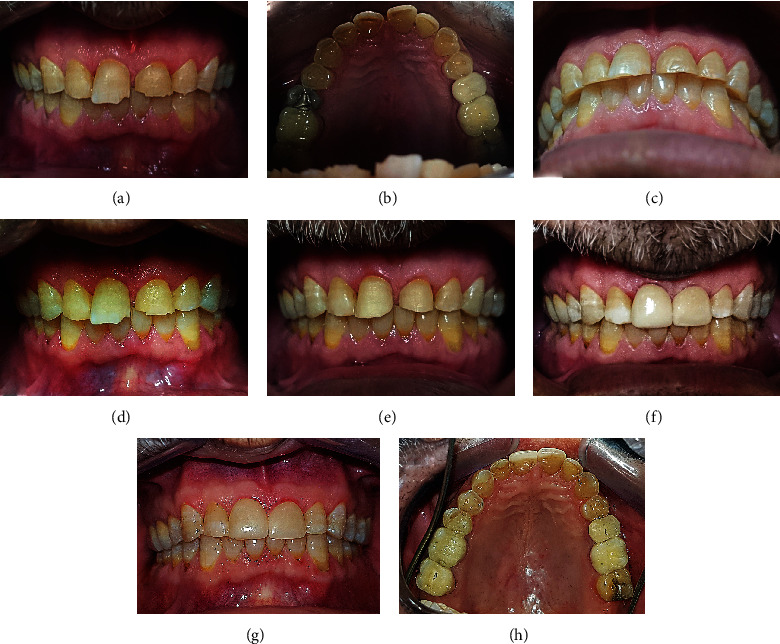
The third case of localized dental wear: (a)–(c), before treatment; (d), after the canine rise method; (e), teeth preparation for two ceramic veneers; (f), after cementation of two veneers; (g) and (h), frontal and occlusal views after three years.

**Figure 6:**
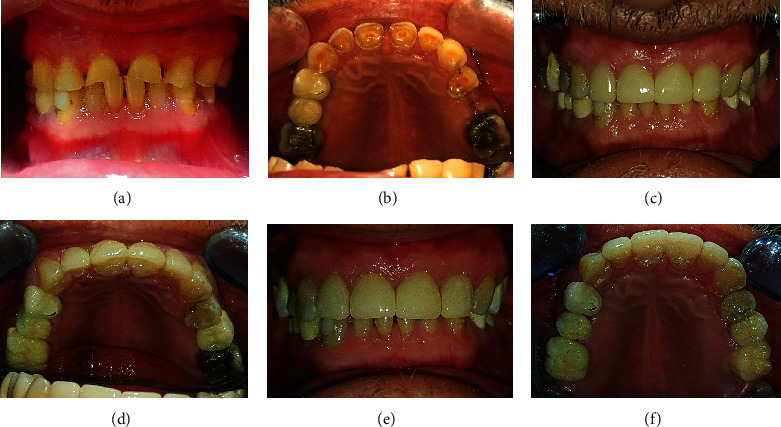
The fourth case of localized dental wear: (a) and (b), before treatment; (c) and (d), the one-year follow-up shows that the extracted upper left premolar area was restored by a dental implant and PFM crown restoration by another practitioner. (e) and (f); frontal and occlusal views of the case after four years.

The other four cases with localized dental wear were treated by the same procedure (canine rise method). Permanent restorations were placed on the canines using composite resin, and the other anterior teeth were restored with either direct composite veneers or indirect ceramic veneers (feldspathic porcelain, Noritake, Kuraray Dental Inc.). These cases were followed up from 2 to 5 years, as shown in Figures 3, 4, 5, and 6. Figures 3(a)–3(c) show severe localized dental wear on the anterior segment of the first case with localized dental wear. The treatment of this patient's worn teeth was started by direct build-up composite resin restorations on 13, 12, 11, 21, 22, and 23, without considering the canine rise method as seen in Figure 3(d). After about two years, the crown on 21 was fractured, as shown in Figures 3(e) and 3(f). Based on canine rise method, a proper space was created to restore 13, 12, 11, 21, 22, and 23 by direct composite restorations without increasing vertical dimension of occlusion as seen in Figures 3(g)–3(i). The occlusion in laterotrusive and protrusive movements showed the canine and anterior teeth guidance, respectively, as seen in Figures 3(i)–3(k). Follow-up of the case after five years is shown in Figures 3(l)–3(n). The second case with localized dental wear on anterior teeth was also treated based on the canine rise method with a direct composite restoration, as seen in Figures 4(a)–4(k). The definitive treatment after 2 years of follow-up is shown in Figures 4(i)–4(m), while the lateral extortion movement is seen in Figures 4(n) and 4(o). Also, after 2 years, composite restoration of left central incisor was completely debonded and restored by new composite restoration as shown in Figures 4(p)–4(r). The third case of localized dental wear was also treated based on the canine rise method with direct composite resin restorations for 13 and 23 and porcelain laminate veneers for 11 and 21, as seen in Figures 5(a)–5(e). The follow-up of the patient after four years is shown in Figures 5(f)–5(h). The fourth case of localized dental wear is shown in Figures 6(a)–6(e). The treatment of the patient's dental wear was started by direct composite resin restoration on the 13 and 23 based on the canine rise method. After one month, porcelain laminate veneers were placed on the remaining 12 and 22 without restoration of posterior teeth, as seen in Figures 6(b) and 6(d). The follow-up of the patient after four years shows that the extracted tooth number 35 has been replaced by a dental implant and PFM crown restoration as seen in Figure 6(e).

## 3. Discussion

Frequently, the occlusal disease remains undiagnosed until severe damage becomes esthetically and functionally discomforting for the patient. It is one of the contributing factors in tooth loss. Treatment planning can be preventive (i.e., controlling the erosive factor), restorative, or a combination. To describe an occlusal treatment as a successful one, it should provide a comfortable and stable TMJ position and centric relation; the anterior teeth must be in harmony with the envelope of function without premature contacts in posterior teeth. The position that allows for interference-free contact is the centric relation; this position is an important factor in treatment planning for dental wear, and similarly, anterior guidance is an important factor to be considered [2].

Loss and fracture of posterior restorations has a multifactorial etiology, including loss of anterior guidance and canine protection. The latter increases horizontal stresses on the posterior occlusal surfaces [17]. These problems are usual with dental wear patients since the loss of the guidance and protection mechanism is usually associated with this type of occlusal disease. The treatment for dental wear and associated side effects basically depends on the type of restoration, the guidance, and the protection mechanism. The use of direct composite restorations to restore the worn-away dental surfaces is a conservative and easy-to-maintain method [20].

The most important question during clinical decision making is how to create space for the restorative material. In the literature, five methods are described to create the spaces needed for restorative materials: (1) preparation of worn teeth (with the same intercuspation position of the posterior teeth; usually, crown lengthening is needed); (2) increasing the vertical dimension of occlusion (VDO) can be used in localized dental wear (DahI technique) or generalized dental wear. The tooth will be restored by either an additive method or a destructive method; (3) tooth movement by orthodontic forces or orthognathic surgeries (in limited cases); (4) distalization by occlusal adjustment of defective contact on posterior teeth (in limited cases); and (5) canine rise method ( see Table 1) [21]. The canine rise method is a conservative approach for restoring the worn teeth in occlusal disease. It is applicable in many cases of worn teeth; also, it is an opportunity for the operator to restore generalized worn teeth (without an articulator and a technician) in a conservative, accurate, inexpensive, and fast way, as seen in Figure 2. Regardless to vertical dimension, the canine rise method is not suggested in class lll, because canine tooth does not have the guidance. However, in cases such as case no. 2 (full-mouth rehabilitation), to reduce chair time, taking the impression to prepare a silicone matrix or clear stand for the build-up of the affected teeth can be another way. Obviously, canine rise must be implemented before taking the impression (case no. 1 in the temporary stage). This method is indicated for patients with occlusion types Cl I and Cl II Angle's classification (except Cl III due to canine tooth does not have the guidance), where restoring worn teeth can be carried out without increasing the vertical dimension, as shown in Figures 1–6. A healthy periodontium of canine teeth with sufficient tooth structure is critical for this method. It is well-established that canine rise occlusion is superior to group function occlusion in providing better protection against nonphysiologic muscle tension [9]. The canine rise method is based on the biofeedback mechanism through periodontal proprioceptive fibers surrounding the root of the canine teeth [22]. These fibers provide muscle function regulatory impulses during lateral excursion movements; more specifically, when canine teeth come in contact, the ratio of shear forces exerted on the canine teeth is regulated by that mechanism through translating the shear forces to the regulatory impulse that, in turn, is fed to the mesencephalic root of the trigeminal nerve, altering the motor impulses to the musculature [23]. Manns et al. observed that the fewer the occlusal contacts, the lower the amount of elevator muscle activity; on the contrary, more occlusal contact points resulted in higher activity [9]. Based on the previous description, the canine rise method decreased muscle tension in patients with this condition. Moreover, it reduced the tonicity of elevator muscles to provide more freedom for the mandible to slide backward. This mandibular movement provides enough space in the anterior dental region and some of the posterior parts, for example, case no. 3 and 4 shown in Figures 3(a)–3(l) and 4(o) and 4(p). In this way, the worn teeth can be restored without any change in the length of upper and lower posterior teeth, creating an ideal condition for the rehabilitation of worn teeth [2]. This method has the following advantages: (1) it is based on the neuromuscular system; (2) anterior worn teeth can be restored without any change in the cuspal contact of posterior teeth; and (3) finishing the restoration of worn teeth is possible without an articulator.

**Figure 7:**
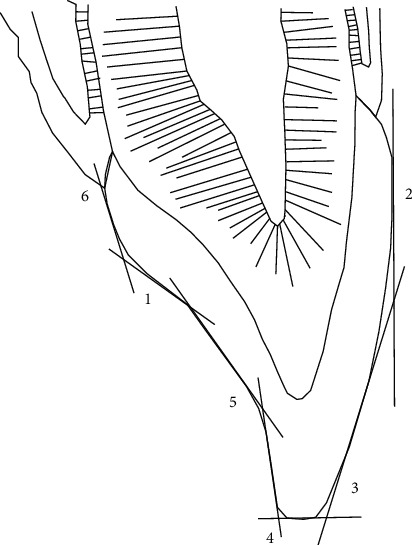
The six factors that determine labial and lingual contour and relate them to the correct incisal edge position: 1, mandible-to-maxilla relationship; 2, lip support; 3, lip closure path; 4, tooth-to-lip relationship; 5, the envelope of function; 6, tooth-to-tooth relationship.

Based on EMG studies, composite restorations in worn canine teeth serve as a canine ramp in an occlusal splint and decrease muscle activity during excursions [24–26]. Furthermore, composite restorations are more comfortable for the patients. To restore worn anterior teeth accurately, as shown in Figures 1to 6, the operator can use clinical judgment instead of using an articulator. As Figure 7 shows, all the anterior teeth, including canines, might be involved in one or a combination of six tooth segments in the wear process: mandible-to-maxilla relationship in centric relation depends on restoring segment 1; restoring segment 2 is important for lip support, and restoring segments 3, 4, 5, and 6 are essential for lip closure path, tooth-to-lip relationship during pronouncing F and V sounds, envelope of function, and tooth-to-tooth relationship while pronouncing the S sound, respectively [2].

The articulator is important only for the reconstruction of segment 1, while dynamic, esthetic, and phonetic factors are critical determinants for restoring the labial/lingual contour in other segments. Since it is unlikely for canine teeth to undergo wear in the segment 1, considering the unique role of these teeth in occlusion and reconstruction of worn teeth, in the first step of the canine rise method, canines should be restored with a direct composite resin procedure. However, if segment 1 of canine teeth is affected by wear, it will be necessary to use an articulator to restore them.

## 4. Conclusion

This report made an effort to present generalized and localized tooth wear rehabilitation with a new method. The images of the patients before and after treatment confirm clinical success. This method is not time-consuming; instead, it is economical and extremely conservative compared to conventional prosthodontic methods. The most important factor in achieving satisfactory clinical results is the ability and experience of the operator. Furthermore, in this method, the presence of a canine tooth with sufficient structure and periodontal support is a key factor. Canine is more resistant to wear than other anterior teeth because of its dome shape of the incisal edge. In our opinion, canine teeth must be considered as a determinant factor for the limitation and guidance of eccentric jaw movements. Increasing the length of canines in this method restricts the occlusal contacts to the canine teeth in eccentric jaw movements and produces adequate space for restorative materials to be added to the other worn teeth with the same intercuspation position of the posterior teeth. In this way, friction and wear of teeth will be reduced.

## Figures and Tables

**Table 1:** Techniques for creating space to allow the restoration of worn teeth.

Technique	Advantages	Disadvantages
Space creation by distalization of the mandible technique [24].	(i) No (or little) anterior tooth reduction required (ii) Can enable worn teeth to be restored without increasing the vertical dimension	Limited to cases where large horizontal RCP-ICP (retruded contact position-inter cuspal position) slide exists
Space creation by increase the vertical dimension technique [24].	No need for an occlusal reduction; therefore, full axial wall length of preparation can be maintained	All teeth in one or both arches may need restorations
Canine rise method	(i) Conservative and not destructive to teeth (ii) Worn teeth can be restored without an increase in the vertical dimension of occlusion (iii) It is possible to finish the restoration of the worn teeth without articulator devices (iv) Because of neuromuscular regulation, it is possible to finish the treatment in a shorter duration	Monitoring the restored canine teeth is essential because losing the restoration may result in damaging of other anterior restored teeth.

## Data Availability

No data were used to support this study.
